# Snoring is associated with hypertension and diabetes mellitus among adults in north Sudan: a cross-sectional study

**DOI:** 10.1186/s12889-024-18505-x

**Published:** 2024-04-08

**Authors:** Amal O. Bashir, Mousab A. Elimam, Mohamed A. Elimam, Ishag Adam

**Affiliations:** 1 Faculty of Public Health and Health Informatics, University of Umm Al Qura, Mekkah, Saudi Arabia; 2 Faculty of Medicine, Alzaiem Alazhari University, Khartoum North, Sudan; 3 Department of Obstetrics and Gynecology, College of Medicine, Qassim University, Buraidah, Saudi Arabia

**Keywords:** Snoring, Obesity, Age, Hypertension, Diabetes mellitus, Females

## Abstract

**Background:**

Different levels of association between snoring, hypertension, and diabetes mellitus (DM) are reported. There are few published studies on this topic in African countries, and no investigation was conducted in Sudan. This study aimed to assess the prevalence and factors associated with snoring and the association between snoring, hypertension, and type 2 DM (T2DM) in northern Sudan.

**Methods:**

A community-based cross-sectional study using a multistage sampling technique was conducted in four villages in the River Nile state of northern Sudan from July to September 2021. Sociodemographic characteristics were collected using a questionnaire. Body mass index (BMI) was measured using standard methods, and a multivariate analysis was conducted using the Statistical Package for the Social Sciences® (SPSS®) for Windows, version 22.0.

**Results:**

Of the 384 adults, 193 (50.3%) were males and 191 (49.7%) were females. Of the adults, 38 (9.9%) were underweight, 121 (31.5%) had average weight, 113 (29.4%) were overweight, and 112 (29.2%) were obese. One hundred and six (27.6%) adults were snorers. Multivariate analysis showed that increasing age (adjusted odds ratio [AOR] = 1.02, 95% confidence interval [CI] = 1.01‒1.04), increasing BMI (AOR = 1.04, 95 CI = 1.01‒1.08), obesity (AOR = 2.0, 95% CI = 1.10‒3.69), and alcohol consumption (AOR = 2.32, 95% CI = 1.14‒4.74) were positively associated with snoring. Of the 384 adults, 215 (56.0%) had hypertension. Multivariate analysis showed that increasing age (AOR = 1.04, 95% CI = 1.02‒1.06), increasing BMI (AOR = 1.08, 95% CI = 1.04‒1.13), female sex (AOR = 1.7, 95% CI = 1.08‒2.73), and snoring (AOR = 1.69, 95% CI = 1.02‒2.82) were positively associated with hypertension. One hundred and six (27.6%) adults had T2DM. Multivariate analysis showed that increasing age (AOR = 1.03, 95% CI = 1.01‒1.05) and snoring (AOR = 1.78, 95% CI = 1.09‒2.91) were associated with T2DM.

**Conclusion:**

Around one-fourth of the adults in Northern Sudan are snorers. Snoring is more common among obese adults. Snoring is associated with increased odds of hypertension and T2DM. Adults who snore must pay close attention to their blood pressure and blood glucose levels to prevent hypertension and DM.

## Introduction

Snoring is an audible respiratory phenomenon that manifests predominantly during sleep and may transpire during nocturnal or diurnal rest periods [1]. Snoring represents a prevalent sleep-related issue with a global impact, affecting a substantial portion of the population [2, 3]. Despite its frequent characterization as benign or humorous, snoring can negatively affect an individual's well-being [2, 3]. Snoring occurs from the oscillation of upper airway structures during sleep. This generates acoustic disturbances as air traverses the respiratory passages during inhalation and exhalation [4]. Previous studies have shown different prevalence of snoring in different populations [5–7]. Snoring demonstrates a substantial prevalence within the population, displaying an age-dependent rise until the seventh decade of life, followed by a modest decline with a discernible sex disparity, affecting a higher proportion of males (35‒45%) than females (15‒28%) [4].

Several factors, such as age, sex, [7] body mass index (BMI) snorers [7–9], alcohol consumption, and cigarette smoking, are associated with habitual snoring [4, 10]. Emerging evidence suggests a bidirectional relationship between snoring and obesity. Adiposity amplifies susceptibility to snoring, while snoring, in turn, augments the risk of metabolic disorders, diabetes mellitus (DM), diminished sleep quality, and daytime drowsiness [8]. Previous studies have scrutinized the heightened incidence of obesity, cardiovascular morbidities, mortality, and metabolic disorders among snorers [8, 11].

Snoring is further associated with elevated risks of overall cardiovascular disease, ischemic heart disease, and ischemic stroke [10, 12–14]. Several previous studies have shown different levels of association between snoring, hypertension, and DM [6, 9, 10, 12–16]. However, few studies on snoring have been conducted in Africa [5, 6]**,** and none of them have been conducted in Sudan, the third-largest African country. It has been shown that 40.8% and 30.8% of Sudanese adults had hypertension and DM, respectively [17, 18]. Research on snoring and its association with hypertension and DM could yield data that could help physicians and healthcare providers apply evidence-based interventions in the community. This study aimed to assess the prevalence and factors associated with snoring and the association between snoring, hypertension, and type 2 DM (T2DM) in northern Sudan.

## Materials and methods

### Study area and sampling technique

A community-based cross-sectional study using a multistage sampling technique was conducted in northern Sudan from July to September 2021 in the River Nile state, one of the 18 states of Sudan. Based on the 2008 census, the state's total population was 1,120,441 [19]. Of the seven localities, Wad Hamid was randomly selected from the seven localities (the lowest administrative units in Sudan) in the River Nile state. Four villages (Hajer Alteer, Athawra Kabota, Wadi Alshohda, and Alkoumer) were randomly selected, out of which 135 households were randomly selected for the study. The number of households depended on the population of the selected village. The Strengthening the Reporting of Observational Studies in Epidemiology (STROBE) guidelines were strictly followed [20].

### Study population and design

All the adults (male and female) in the selected household who agreed to participate and met the study inclusion and exclusion criteria were enrolled. The next house was selected to enroll the adults when the chosen house was uninhabited.

### Inclusion and exclusion criteria

Adults (males and females) 18 years of age or older who signed an informed consent form and were Sudanese residents were included in the study. Participants under 18 years of age who refused to give consent, pregnant women, those with type 1 DM, patients with poor cognitive functions, and severely ill patients were excluded from this study.

### Data collection

The World Health Organization's (WHO) three-level stepwise approach questionnaire was used to collect the data for this study [21]. The questionnaire was used to collect data on sociodemographic characteristics, including the adults' age, sex (male or female), employment status (employed or non-employed), marital status (married or unmarried), education level (< secondary and ≥ secondary), smoking of cigarettes (never or former/current), alcohol consumption (never or former/current), and family history of DM and hypertension. The questionnaire and the clinical examinations were conducted through face-to-face interviews with two trained medical officers (male and female). The principal investigator trained the medical officers for two days on the data collection and the clinical examinations.

### Procedures

After resting for 10 min sitting, blood pressure was measured twice using an appropriate cuff size with a standard mercury sphygmomanometer. The mean of the two readings was computed, and when the difference between the two readings exceeded five mmHg, a third measurement was taken. Adults were considered to have hypertension if the systolic blood pressure was ≥ 140 mmHg, the diastolic blood pressure was ≥ 90 mmHg, both criteria were met in the repeated measurements, or the use of antihypertensive medications for high blood pressure was reported [22].

Adults` weight and height were measured twice using standard procedures, and the mean of the two readings was calculated. Weight was measured in kg when the adults stood with minimal movement, their hands by their sides, and their shoes and excess clothing removed. Height was measured in cm while participants stood straight with their backs against the wall and feet together. The weight and height measurements were used to calculate BMI using the equation of weight in kg divided by the square of the height in meters (kg/m^2^). The WHO classification of BMI was applied. Underweight was defined when BMI < 18.5kg/m^2^), average weight when BMI was 18.5–24.9 kg/m^2^, overweight when BMI was 25.0–29.9 (kg/m^2^), and obese when BMI was ≥ 30.0 kg/m^2^) [23].

Certified lab technicians withdrew 3–5 ml of blood from each participant for testing HBA1c using an Ichroma machine following the manufacturer's instructions (Chuncheon-si, Gang-won-do, 24398, Republic of Korea), as described in our previous work [24].

### Diabetes mellitus

A diagnosis of DM was considered for those who had documentation of T2DM. The goal was to determine whether they were on diet control or glucose-lowering drugs during the study period or met the American Diabetes Association's diagnosis of DM for non-pregnant adults and definition by the International Diabetes Federation [25, 26]: random plasma glucose levels of ≥ 200 mg/dl (11.1 mmol/L) in patients with classic symptoms of hyperglycemia (polydipsia, polyuria, and polyphagia) or hyperglycemic crisis or glycated hemoglobin level of ≥ 6.5% was the primary diagnostic criterion.

### Measurement of snoring

Adults were asked, 'Have you been told that you are snoring when you are asleep?' If the adults answered yes, they were asked about the frequency of snoring [27].

### Sample size calculation

A sample size of 384 adults was calculated using OpenEpi Menu [28], and it was based on the assumption of the expected prevalence (50.0%) of snoring among the adults. The assumed prevalence of snoring depended on the prevalence (59.9%) of snoring previously reported in one African country (Cameron) [6]. Then, we assumed that 40.0% of the snorers would be obese and 25.0% of the non-snorers would be obese. The assumption of the prevalence of obesity was based on the previous prevalence of obesity in eastern Sudan [29]. The sample size for the second objective (the association between snoring and hypertension) was based on the prevalence of hypertension (40.8%) in eastern Sudan [17]. The sample size of 384 adults was calculated to detect a precision e of 5% at α = 0.05, with a power of 80%.

### Statistical analysis

**Fig. 1:**
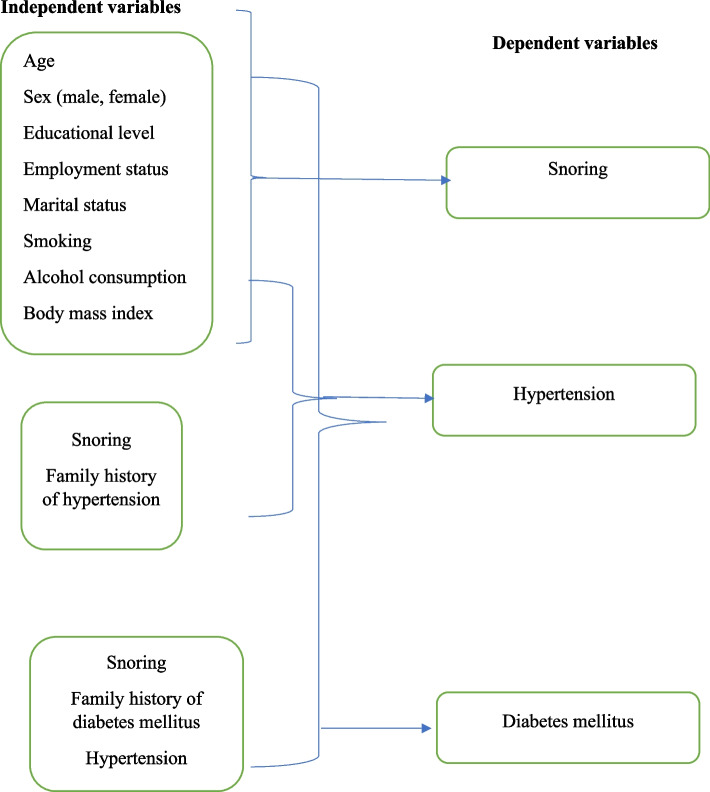
Flow chart, dependent and independents variables for snoring, hypertension diabetes mellitus in Northern Sudan (number = 384), 2022

The collected data were checked, entered into an Excel sheet, cleaned, edited, and then exported to The Statistical Package for the Social Sciences® (SPSS®) for Windows, version 22.0 (SPSS Inc., New York, United States) for analysis. The proportions were expressed as frequencies (%) and compared between the two groups using the Chi-square test. The Shapiro–Wilk test was used to evaluate the continuous data for normality and were non-normally distributed. The results were expressed as the median interquartile range (IQR). A univariate analysis was conducted in which snoring, hypertension, and T2DM were the dependent variables. The independent variables were sex, BMI, educational level, occupation, marital status, smoking, alcohol consumption, family history of hypertension (when hypertension was the dependent variable), family history of DM (when T2DM was the dependent variable), hypertension and snoring (when hypertension and T2DM were the dependent variables). Variables with *P* < 0.05 were shifted to expand the binary multivariate analysis to rule out the confounders (Fig. 1). The Variance Inflation Factor (VIF) was used to check multicollinearity in regression analysis and it was not detected. Hosmer—Lemeshow test was used to check the model's adequacy fitness, and it was found that the model fit the data adequately. Adjusted odds ratios (AORs) with 95% confidence intervals (CIs) were calculated as they were applied. A two-sided *P*-value of < 0.05 was considered statistically significant.

## Results

### General characteristics

Three hundred eighty-four adults were enrolled, of whom 193 (50.3%) were male and 191 (49.7%) were female. The median (IQR) of the adults` age was 45.0 (33.0‒55.0) years. Of the 384 adults, 258 (67.2%) and 126 (32.8%) had their education level ≥ secondary (8 years of education) and < secondary, respectively. Of the adults, 38 (9.9%) were underweight, 121 (31.5%) had normal weight, 113 (29.4%) were overweight, and 112 (29.2%) were obese. The details of the adults' characteristics are shown in Table 1.

**Table 1:** General characteristics of the adults in Northern Sudan (number = 384), 2022

Age, years		Range (Interquartile range)
		45.0(33.0‒55.0)
Body mass index, kg/m^2^		26.4(22.4‒30.7)
		Frequency (Proportion)
Sex	Male	193(50.3)
	Female	191(49.7)
Education level	≥ secondary	258(67.2)
	< secondary	126(32.8)
Occupational status	Employed	178(46.4)
	No-employed	206(53.6)
Marital status	Married	96(25.0)
	Unmarried	288(75.0)
Cigarette smoking	Never	305(79.4)
	Current/former	79(20.6)
Alcohol consumption	Never	345(89.8)
	Current/former	39(10.2)
Family history of hypertension	No	193(50.3)
	Yes	191(49.7)
Family history of diabetes mellitus	No	246(64.1)
	Yes	138(35.9)
Snoring	No	278(72.4)
	Yes	106(27.6)
Body mass index, kg/m^2^	Underweight	38(9.9)
	Normal	121(31.5)
	Overweight	113(29.4)
	Obese	112(29.2)

### Factors associated with snoring

One hundred and six (27.6%) adults were snorers (9.1%, 12.0%, and 6.5% had snoring almost always, often, and sometimes, respectively). In univariate analysis, increasing age, BMI, obesity, marital status, and alcohol consumption were positively associated with snoring. Sex, education, occupation, smoking, and obesity were not associated with snoring. Multivariate analysis showed that increasing age (AOR = 1.02, 95% CI = 1.01‒1.04, *P* = 0.002), increasing BMI (AOR = 1.04, 95 CI = 1.01‒1.08, *P* = 0.021), obesity (AOR = 2.0, 95% CI = 1.10‒3.69, *P* = 0.023), and alcohol consumption (AOR = 2.32, 95% CI = 1.14‒4.74, *P* = 0.020) were positively associated with snoring. Thus, marital status was associated with snoring only in the univariate analysis (confounder) (Table 2).

**Table 2:** Univariate and multivariate analysis of the factors associated with snoring among adults in Northern Sudan, 2022

Variable		Participants with snoring (number = 106)	Participants without snoring (number = 278)	Univariate analysis		Multivariate analysis	
Range (Interquartile range)				OR (95% CI)	*P*	OR (95% CI)	*P*
Age, years		51.5(42.0‒59.2)	42.0(30.0‒55.0)	1.03(1.01‒1.04)	< 0.001	1.02(1.01‒1.04)	0.002
Body mass index, kg/m^2^		27.5(23.5‒31.9)	25.7(21.9‒30.1)	1.05(1.01‒1.08)	0.008	1.04(1.01‒1.08)	0.021
Frequency (Proportion)							
Sex	Male	55(51.9)	138(49.6)	Reference			
	Female	51(48.1)	140(50.4)	0.91(0.58‒1.43)	0.694		
Education level	≥ secondary	74(69.8)	184(66.2)	Reference		‒	-
	< secondary	32(30.2)	94(33.8)	0.84(0.52‒1.37)	0.499	‒	
Occupational status	Employed	50(47.2)	128(46.0)	Reference			
	No-employed	56(52.8)	150(54.0)	0.95(0.61‒1.49)	0.843	‒	
Marital status	Married	17(16.0)	79(28.4)	Reference			0.345
	Unmarried	89(84.0)	199(71.6)	1.56(0.96‒2.51)	0.07	1.35(0.72‒2.53)	
Cigarette smoking	Never	88(83.0)	217(78.1)	Reference			
	Current/former	18(17.0)	61(21.9)	0.72(0.40‒1.30)	0.284	‒	
Alcohol consumption	Never	89(84.0)	256(92.1)	Reference			0.020
	Current/former	17(16.0)	22(7.9)	2.22(1.12‒4.37)	0.021	2.32(1.14‒4.74)	
Body mass index, kg/m^2^	Underweight	7(6.6)	31(11.2)	0.82(0.32‒2.08)	0.685	0.81(0.31‒2.13)	0.677
	Normal	26(24.5)	95(34.2)	Reference		Reference	
	Overweight	33(31.1)	80(28.2)	1.50(0.83‒2.72)	0.176	1.54 (0.83‒2.86)	0.166
	Obese	40(37.7)	72(25.9)	2.03(1.13‒3.62)	0.017	2.01(1.10‒3.69)	0.023

### Factors associated with hypertension

Two hundred and fifteen (56.0%) adults had hypertension (38.8% and 17.2% of adults had newly diagnosed hypertension and were known to have hypertension, respectively). Seventy-two of the 215 (33.5%) hypertensive adults and 34 of the 169 (20.1%) of the non-hypertensive adults were snorers (*P* = 0.003). In the univariate analysis, increasing age, female sex, BMI, overweight, obesity, marital status, family history of hypertension, alcohol consumption, and snoring were positively associated with hypertension. Education, occupation, and smoking were not associated with hypertension. Multivariate analysis showed that increasing age (AOR = 1.04, 95% CI = 1.02‒1.06, *P* < 0.001), increasing BMI (AOR = 1.08, 95% CI = 1.04‒1.13, *P* < 0.001), female sex (AOR = 1.7, 95% CI = 1.08‒2.73, *P* = 0.022), and snoring (AOR = 1.69, 95% CI = 1.02‒2.82, *P* = 0.041) were positively associated with hypertension (Table 3).

**Table 3:** Univariate and multivariate analysis of the factors associated with hypertension among adults in Northern Sudan, 2022

Variable		Participants with hypertension (number = 215)	Participants without hypertension (number = 169)	Univariate analysis		Multivariate analysis	
Range (Interquartile range)				OR (95% CI)	*P*	OR (95% CI)	*P*
Age, years		50.0(38.0‒60.0)	38.0(28.0‒50.0)	1.04(1.02‒1.05)	< 0.001	1.04(1.02‒1.06)	< 0.001
Body mass index, kg/m^2^		27.7(24.8‒31.4)	24.4(19.5‒28.5)	1.10(1.06‒1.14)	< 0.001	1.08(1.04‒1.13)	< 0.001
Frequency (Proportion)							
Sex	Male	95(44.2)	98(58.0)	Reference		Reference	0.022
	Female	120(55.8)	71(42.0)	1.74(1.16‒2.62)	0.007	1.71(1.08‒2.73)	
Education level	≥ secondary	145(67.4)	113(66.9)	Reference			
	< secondary	70(32.6)	56(33.1)	0.97(0.63‒1.49)	0.905	‒	
Occupational status	Employed	92(42.8)	86(50.9)	Reference			
	No-employed	123(57.2)	83(49.1)	1.38(0.92‒2.07)	0.115	‒	
Marital status	Married	45(20.9)	51(30.2)	Reference			0.332
	Unmarried	170(79.1)	118(69.8)	1.63(1.02‒2.59)	0.039	0.75(0.42‒1.33)	
Cigarette smoking	Never	176(81.9)	129(76.3)	Reference			
	Current/former	39(18.1)	40(23.7)	0.71(0.43‒1.17)	0.184		
Alcohol consumption	Never	197(91.6)	148(87.6)	Reference			
	Current/former	18(8.4)	21(12.4)	0.64(0.33‒1.25)	0.194		
Family history of hypertension	No	91(42.3)	102(60.4)	Reference		Reference	0.090
	Yes	124(57.3)	67(39.4)	2.07(1.37‒3.12)	< 0.001	1.49(0.94‒2.36)	
Snoring	No	143(66.5)	135(79.9)	Reference		Reference	0.041
	Yes	72(33.5)	34(20.1)	1.99(1.24‒3.20)	0.004	1.69(1.02‒2.82)	
Body mass index, kg/m^2^	Underweight	5(2.3)	33(19.5)	0.14(0.05‒0.39)	< 0.001	0. 16(0.05‒0.46)	0.001
	Normal	62(28.8)	59(34.9)	Reference		Reference	
	Overweight	74(34.4)	39(23.1)	1.80(1.06‒3.05)	0.028	1.66(0.95‒2.92)	0.075
	Obese	74(34.4)	38(22.5)	1.85(1.09‒3.14)	0.022	1.62(0.92‒2.87)	0.093

### Factors associated with type 2 diabetes mellitus

One hundred and six (27.6%) of the 384 adults had T2DM (16.4% and 11.2% had newly diagnosed T2DM and were known to have T2DM, respectively). Forty of the 106 (37.7%) adults with T2DM versus 66 of 278 (23.7%) (*P* = 0.006) of the adults without T2DM were snorers. In the univariate analysis, increasing age, marital status, family history of T2DM, and snoring were positively associated with T2DM. Female sex, increasing BMI, overweight, obesity, education, occupation, smoking, and alcohol consumption were not positively associated with T2DM. The multivariate analysis showed that increasing age (AOR = 1.03, 95% CI = 1.01‒1.05, *P* < 0.001) and snoring (AOR = 1.78, 95% CI = 1.09‒2.91, *P* = 0.020) were associated with T2DM (Table 4).

**Table 4:** Univariate and multivariate analysis of the factors associated with types 2 diabetes mellitus among adults in Northern Sudan, 2022

Variable		Participants with diabetes mellitus (number = 106)	Participants without diabetes mellitus (number = 278)	Univariate analysis		Multivariate analysis	
Range (Interquartile range)				OR (95% CI)	*P*	OR (95% CI)	*P*
Age, years		52.5(42.7‒60.0)	40.0(30.0‒53.2)	1.04(1.02‒1.05)	< 0.001	1.03(1.01‒1.05)	< 0.001
Body mass index, kg/m^2^		27.1(23.5‒31.0)	25.8(22.0‒30.7)	1.02(0.98‒1.06)	0.175		
Frequency (Proportion)							
Sex	Male	45(42.5)	148(53.2)	Reference			
	Female	61(57.5)	130(46.8)	1.54(0.98‒2.42)	0.060		
Education level	≥ secondary	70(66.0)	188(67.6)	Reference			
	< secondary	36(34.0)	90(32.4)	1.07 (0.66‒1.72)	0.767	‒	
Occupational status	Employed	45(42.5)	133(47.8)	Reference			
	No-employed	61(57.5)	145(52.2)	1.24(0.79‒1.95)	0.344	‒	
Marital status	Married	16(15.1)	80(28.8)	Reference			0.306
	Unmarried	90(84.9)	198(71.2)	2.27(1.25‒4.10)	0.007	1.39(0.73‒2.63)	
Cigarette smoking	Never	90(84.9)	215(77.3)	Reference			
	Current/former	16(15.1)	63(22.7)	0.60(0.33‒1.10)	0.103		
Alcohol consumption	Never	99(934)	246(88.5)	Reference			
	Current/former	7(6.6)	32(11.5)	0.54(0.23‒1.27)	0.160		
Family history of diabetes mellitus	No	60(56.6)	78(28.1)	Reference		Reference	
	Yes	46(43.4)	200(71.9)	3.34(2.10‒5.32)	< 0.001	0.99(0.62‒1.59)	
Snoring	No	66(62.3)	212(76.3)	Reference			0.020
	Yes	40(37.7)	66(23.7)	1.94(1.20‒3.14)	0.007	1.78(1.09‒2.91)	
Body mass index, kg/m^2^	Underweight	4(3.8)	34(12.2)	0.34(0.11‒1.04)	0.059		
	Normal	31(29.2)	90(32.4)	Reference			
	Overweight	40(37.7)	73(26.3)	1.59(0.90‒2.78)	0.105		
	Obese	31(29.2)	81(29.1)	1.11(0.62‒1.98)	0.722		

### Factors associated with both hypertension and type 2 diabetes mellitus

Seventy-two (18.1%) of the 384 adults had both hypertension and T2DM. The multivariate analysis showed that increasing age (AOR = 1.05, 95% CI = 1.03‒1.07, *P* < 0.001) and snoring (AOR = 2.02, 95% CI = 1.16‒3.51, *P* = 0.013) were associated with both hypertension and T2DM.

## Discussion

This study showed that 27.6% of the adults had snoring associated with increasing age, BMI, and alcohol consumption; snoring increased the odds of hypertension and T2DM. This finding is consistent with the previous study conducted among 843 elderly patients in Nigeria, which showed that in 31.2% of the patients who snored, snoring was significantly associated with obesity [5]. In Cameroon, 59.9% of the patients admitted to the Yaounde Central Hospital's cardiology, endocrinology, and neurology departments snored, and this was significantly higher in men [6]. In Canada, snoring was present in 46.2% of men and 47.0% of women; smoking and obesity were associated with snoring [7]. In India, snorers had double the odds of obesity and seven times the odds of obesity II compared to non-snorers [8]. A meta-analysis of 40 studies with 966,652 participants reported a significant association between obesity and snoring (the pooled ORs = 1.75, 95% CI = 1.46–2.05) [9].

This study demonstrated that snorers had a higher risk of hypertension at 1.69 (AOR = 1.69). Several previous studies have reported an association between snoring and hypertension [10, 13, 14]. In their meta-analysis, Niu. et al. reviewed 11 studies and reported that snoring was associated with an increased risk of hypertension [30]. Moreover, a meta-analysis by Ma et al. that included 40 studies with 966,652 participants reported a significant association between snoring and hypertension, with pooled ORs = 1.23, (95% CI = 1.15–1.31) and 1.75 (95% CI, 1.46–2.05) [9]. While the precise mechanism by which snoring contributes to increased blood pressure is not entirely understood, it is postulated that persistently obstructed breathing during sleep may represent an independent mechanistic pathway through which snoring affects blood pressure. For instance, snoring might impact blood pressure via intrathoracic pressure effects on baroreceptor-mediated control of systemic (and potentially pulmonary) blood pressure or potentially through carotid baroreceptor effects induced by mechanical vibration from the pharyngeal airway [31].

This study found that snorers had a 1.78 (AOR = 1.78) higher risk of T2DM. A study highlighted significant connections between snoring, excessive daytime sleepiness (EDS), and a heightened risk of hypertension and DM in women [14]. Women reporting snoring and EDS exhibited higher rates of hypertension and DM compared to those without these symptoms. Notably, the association between snoring/EDS and hypertension/diabetes remained significant, even after accounting for other risk factors such as age, BMI, smoking, and alcohol consumption. These findings underscore the potential significance of managing sleep disturbances as modifiable risk factors for hypertension and DM in women.

In the Chinese population, the frequency of snoring was positively correlated with the prevalence of type 2 DM (T2DM) [11, 27]. This association was notably more substantial in women and younger age groups than in men and older age cohorts. Another study emphasized that habitual snoring and obesity independently contribute to the risk of developing DM. In particular, men with both factors exhibited a higher risk than those without snoring or having a lower BMI [32]. A meta-analysis by Xiong et al*.,* including eight studies enrolling 101,246 participants, found that snoring was associated with DM (OR = 1.37, 95% CI = 1.20–1.57) [33]. Potential mechanisms included snoring's impact on sleep quality through oxygen desaturation and upper airway obstruction, potentially leading to insulin resistance and glucose metabolism disruption [34]. Furthermore, snoring can disrupt the slumber of the snorer, culminating in sleep deprivation, which, when protracted, can contribute to the development of non-communicable diseases. This deprivation has been linked to conditions such as obesity, insulin resistance, and impaired glucose metabolism [35].

Another significant observation was made in a study in which men with habitual snoring had more than double the incidence of DM compared to non-habitual snorers in the same age group [32]. While obesity largely accounted for the increased risk of diabetes in habitual snorers, it is noteworthy that the odds ratio for diabetes development appeared higher in obese snorers than in obese non-snorers, though not achieving statistical significance. Obstructive sleep apnea, a condition characterized by recurrent breathing interruptions during sleep that often stems from complete or partial airway blockages, is a plausible explanation for the observed association between snoring and non-communicable diseases, e.g., hypertension and DM [4].

These studies highlight the intricate relationships between snoring and the increased risk of hypertension and diabetes, especially in different demographics and with varying contributory factors. The findings emphasize recognizing and managing snoring as a potential risk factor for these health conditions. While snoring is commonly trivialized, it exerts a noteworthy influence on an individual's health. The association between snoring and non-communicable diseases emphasizes the necessity of acknowledging snoring as a potential risk factor. Timely intervention, lifestyle modifications, and, when warranted, treatment can mitigate the impact of snoring on non-communicable diseases, thereby promoting overall health and well-being. Both individuals and healthcare professionals must understand the potential health hazards linked to snoring and take appropriate measures to manage and mitigate these risks [36].

### Limitations of this study

This cross-sectional study might not have detected the precise associations between snoring, hypertension, and T2DM. The duration and intensity of snoring were not measured, other elements of obstructive sleep apnea were not accessed in detail, and further research is needed. Other variables related to metabolic syndrome, such as dyslipidemia, were not investigated and should be considered in future studies.

## Conclusion

Approximately one-third of the adult participants in this study were snorers (especially among the obese), which was positively associated with increased odds for hypertension and T2DM. Adults who snore have to pay close attention to their blood pressure and blood glucose levels to prevent hypertension and DM.

## Data Availability

The data supporting the current study's findings will be available from the corresponding author upon rational request.

## Abbreviations

AORs: Adjusted Odds Ratios
BMI: Body mass index
CI: Confidence Interval
DM: Diabetes mellitus
IQR: Interquartile range
SD: Standard Deviation
SPSS: Statistical Package for Social Science
WHO: World Health Organization
